# Cirsilineol Treatment Attenuates PM_2.5_-Induced Lung Injury in Mice

**DOI:** 10.3390/ijms232213948

**Published:** 2022-11-12

**Authors:** Chaeyeong Kim, Go Oun Kim, Jong-Sup Bae

**Affiliations:** College of Pharmacy, Research Institute of Pharmaceutical Sciences, Kyungpook National University, Daegu 41566, Korea

**Keywords:** cirsilineol, particulate matter, lung toxicity, TLR2, 4–mTOR autophagy, apoptosis

## Abstract

Ultrafine particulate matter with less than 2.5 μm diameter (PM_2.5_) is an air pollutant that causes severe lung damage. Currently, effective treatment and preventive methods for PM_2.5_-induced lung damage are limited. Cirsilineol (CSL) is a small natural compound isolated from *Artemisia vestita*. In this study, the efficacy of CSL on PM_2.5_-induced lung toxicity was tested, and its mechanism was identified. Lung injury was caused by intratracheal administration of PM_2.5_ suspension in animal models. Two days after PM_2.5_ pretreatment, CSL was injected via mouse tail vein for two days. The effects of CSL on PM_2.5_-induced lung damage, autophagy, apoptosis, and pulmonary inflammation in a mouse model and their mechanisms were investigated. CSL significantly suppressed histological lung damage and lung wet/dry weight proportion. CSL also significantly reduced PM_2.5_-induced autophagy dysfunction, apoptosis, lymphocyte suppression, and inflammatory cytokine levels in bronchoalveolar fluid (BALF). Furthermore, CSL increased mammalian target of rapamycin (mTOR) phosphorylation and significantly inhibited the expression of Toll-like receptors (TLR) 2 and 4, MyD88, and the autophagy proteins, Beclin1 and LC3II. Thus, CSL exerts protective effects on pulmonary damage by regulating mTOR and TLR2,4–myD88 autophagy pathways. Therefore, CSL can be used as an effective treatment for PM_2.5_-induced lung damage.

## 1. Introduction

Due to the rapid economic development around the world in recent years, air pollutants have become a leading cause of several pulmonary diseases [1,2]. Ultrafine particulate matter with less than 2.5 μm diameter (PM_2.5_) is the primary air pollutant. Approximately 96% of PM_2.5_ accumulates in the lungs owing to its small size and causes various diseases in the respiratory and circulatory systems [3]. High PM_2.5_ concentrations promote the production and release of inflammatory mediators, which damage lung tissues, leading to respiratory lung injury and inflammation. Eventually, the respiratory disease leads to severe mortality and morbidity [4,5]. Secretion of cytokines and chemokines by PM_2.5_, such as interleukin (IL) and tumor necrosis factor (TNF)-α, causing inflammation. Furthermore, it results in the development of many respiratory diseases, including chronic obstructive pulmonary disease (COPD), acute pulmonary injury, and asthma [6,7]. Thus, a correlation exists between PM_2.5_ exposure and disease-related mortality.

Apoptosis and autophagy are required to maintain lung function. However, uncontrolled autophagy owing to PM_2.5_ exposure induces cell death and causes apoptosis, which advances to pathological pulmonary damage [8]. PM_2.5_-induced cellular oxidative stress can induce apoptosis and autophagy. Furthermore, PM_2.5_ damages cellular components and inhibits the activation of mammalian target rapamycin (mTOR), a tropic sensor [9,10]. Autophagy, a lysosomal-dependent process, is involved in adaptation, protein aggregation, and organ damage to pathogen rotations within cells [11]. Autophagy is involved not only in the disease itself but also in lung inflammation observed in conditions such as COPD and lung injury [12,13].

Traditional Chinese and African herbal medicines have helped treat various ailments, including stroke, cardiovascular disease, diabetes, inflammatory disorders, and liver diseases; however, these benefits lack scientific validation [14,15]. Research on the application of traditional herbal and botanical medicines are rapidly increasing [15]. Natural products are increasingly preferred owing to their lack of side effects and cost-effectiveness [15]. Several medicinal herbs potently treat lung inflammation [14]. In this study, we evaluated the efficacy of bioactive compounds for treating PM_2.5_-induced pulmonary inflammation. Cirsilineol (CSL), 4,5-dihydroxy-3,6,7-trimethoxyflavone is a flavone bioactive compound present in *Artemisia vestita* Wall, an herb belonging to the Asteraceae family distributed in China and Tibet. CSL is a potent antioxidant, antibacterial, hypnotic, antitumor, and calmative drug, and exhibits cytotoxicity against many cancer cells [16,17,18,19]. However, the pharmacological role of CSL on PM_2.5_-induced lung injury, inflammatory response, histological changes, and the TLR-autophagy pathway remains unelucidated. Thus, in this study, we investigated the effect of CSL on autophagy and inflammation in cellular and animal models after PM_2.5_ exposure and demonstrated that CSL improved the repair of PM_2.5_-induced tissue damage.

## 2. Results

### 2.1. CSL Protects against PM_2.5_-Induced Lung Toxicity

**Figure 1:**
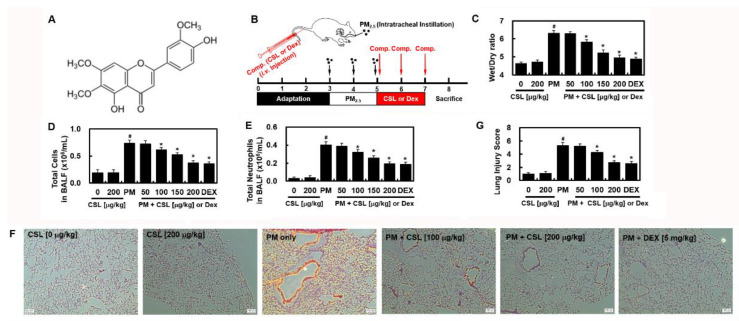
Effects of cirsilineol (CSL) on PM_2.5_-induced lung toxicity. (**A**) Chemical structure of cirsilineol (CSL). (**B**) Schematic illustration of in vivo PM_2.5_-induced lung toxicity experiment protocol. Two days after PM_2.5_ exposure (10 mg/kg in 50 μL saline) daily by intratracheal instillation, the mice were intravenously injected with CSL or dexamethasone (DEX) daily for three consecutive days. The control and PM_2.5_ groups received an equal amount of vehicle [0.5% dimethyl sulfoxide (DMSO)] at the same time each day. Control mice received the same volume of saline. Mice were euthanized 24 h after the last compound administration, and lung tissue and bronchoalveolar lavage fluid (BALF) were harvested. The effects of different CSL or DEX concentrations on (**C**) wet/dry (W/D) weight ratio, (**D**) total cell count in the BALF, and (**E**) total neutrophils in BALF were assessed. (**F**) Lung histology was examined using H&E staining. Representative images from each group are shown (n = 5). Scale bar = 160 μm. (**G**) Lung injury scores. Values represent the mean ± standard deviation (SD) of three independent experiments. * *p* < 0.01 versus the PM_2.5_-challenged group. # *p* < 0.01 versus control group.

Figure 1A represents the chemical structure of CSL. The experiments were conducted according to the workflow in Figure 1B. The mice were administrated with PM_2.5_ for 48 h, and CSL or DEX was injected for another 48 h. After 24 h, the W/D ratio in the PM_2.5_-treated mice increased substantially and was ameliorated by CSL treatment (Figure 1C). Furthermore, total cell and neutrophil count in BALF significantly reduced after CSL injection (Figure 1D,E). Hematoxylin and eosin (H&E) staining confirmed that the lung tissue structure of the control and CSL-treated mice were similar (Figure 1F). Pathological damages such as inflammatory cell infiltration, alveolar hemorrhage, exudates in the alveolar spaces, and alveolar wall thickening were observed in the PM_2.5_-treated mice lung tissue, and these changes were ameliorated by CSL. CSL administration also substantially decreased the lung tissue injury score (Figure 1G). These results indicate that CSL administration alleviated PM_2.5_-induced pulmonary damage.

### 2.2. CSL Prevents PM_2.5_-Induced Autophagy Dysfunction

**Figure 2:**
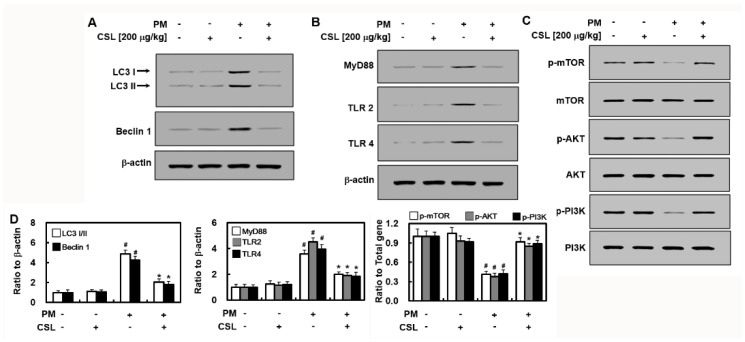
Effects of CSL on PM_2.5_-induced signaling pathways. Representative examples of Western blots showing (**A**) LC3 and Beclin 1, (**B**) Toll-like receptor (TLR) 2, 4, and MyD88, and (**C**) p-mTOR, mTOR, p-Akt, Akt, p-PI3K, and PI3K expression levels. Representative images from each group are shown (n = 3). (**D**) Graphs show densitometric intensities for each gene normalized to that of β-actin or total protein. Values represent the mean ± SD of three independent experiments. * *p* < 0.01 versus the PM_2.5_-challenged group. # *p* < 0.01 versus the control group.

We tested whether CSL affected autophagy-associated proteins, such as Beclin 1 and LC3, by Western blotting. The expression of these autophagy-associated proteins substantially increased in PM_2.5_-treated mice than that in the control mice. Notably, CSL substantially inhibited these increases in mouse lung tissue (Figure 2A). These results suggest that CSL inhibits PM_2.5_-induced autophagy. Subsequently, the expression of proteins in the TLR 2,4 and mTOR autophagy pathways were determined to confirm the CSL-mediated anti-autophagy and anti-inflammatory mechanisms. Intratracheally injected PM_2.5_ increased TLR2, TLR4, and MyD88 expression levels (Figure 2B), which were ameliorated by CSL administration. Moreover, phosphorylated mTOR, Akt, and PI3K expression levels in PM_2.5_-treated mice decreased than that in the control mice (Figure 2C), which were considerably inhibited by CSL treatment. Thus, these results suggest that CSL activates the mTOR/Akt/ PI3K pathway.

### 2.3. CSL Inhibits PM_2.5_-Induced Apoptosis in Mice Lung Tissues

**Figure 3:**
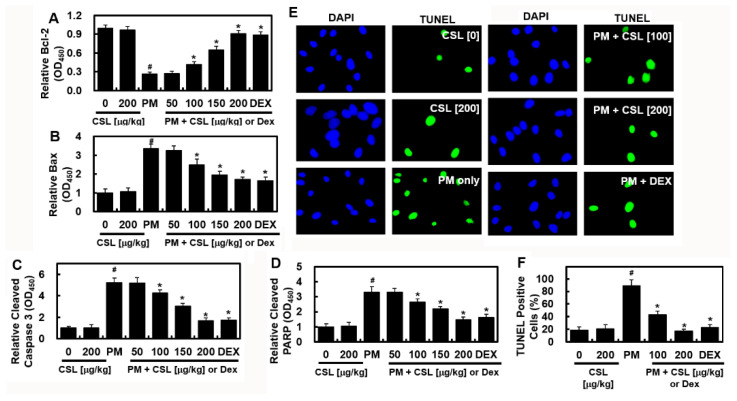
Effects of CSL on PM_2.5_-induced apoptosis. The levels of (**A**) Bcl-2, (**B**) Bax, (**C**) cleaved caspase 3, and (**D**) cleaved PARP were determined using ELISA. (**E**) TUNEL staining of apoptotic cells in lung tissues ×200, and (**F**) the apoptosis ratio. Values represent the mean ± SD of three independent experiments. * *p* < 0.01 versus the PM_2.5_-challenged group. # *p* < 0.01 versus the control group.

To determine whether PM_2.5_ affects apoptosis in lung tissue, the levels of apoptotic proteins, including Bcl-2, Bax, cleaved caspase 3, and cleaved poly [ADP-ribose] polymerase 1 (PARP1), were evaluated in the lung tissue. The level of Bcl-2, an anti-apoptotic protein, was significantly reduced, whereas those of the pro-apoptotic proteins, Bax, cleaved caspase3, and cleaved PARP1, were substantially elevated (Figure 3A–D). However, CSL prevented considerable alterations in these apoptotic protein levels (Figure 3A–D). Consistently, PM_2.5_ treatment considerably elevated the apoptosis index, as demonstrated using TUNEL immunofluorescence (Figure 3E,F). However, CSL treatment markedly reduced the TUNEL apoptosis index, indicating that CSL reduced PM_2.5_-induced apoptosis (Figure 3E,F).

### 2.4. CSL Protected PM_2.5_-Induced Pulmonary Inflammatory Responses in Mice

**Figure 4:**
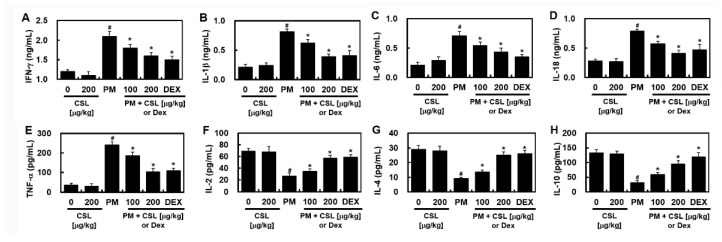
The effects of CSL on PM_2.5_-induced inflammatory cytokine levels. The levels of (**A**) IFN-γ, (**B**) IL-1β, (**C**) IL-6, (**D**) IL-18, (**E**) TNF-α, (**F**) IL-2, (**G**) IL-4, and (**H**) IL-10 were determined by ELISA. Values represent the mean ± SD of three independent experiments. * *p* < 0.01 versus the PM_2.5_-challenged group. # *p* < 0.01 versus the control group.

PM_2.5_ treatment substantially increased pro-inflammatory cytokines such as IFN-γ, IL-1β, IL-6, IL-18, and TNF-α, which were ameliorated by CSL injection (Figure 4). Conversely, the levels of anti-inflammatory cytokines such as IL-2, IL-4, and IL-10 were significantly decreased after PM_2.5_ administration and recovered by CSL injection (Figure 4). These results indicate that CSL can be used to protect mice against PM_2.5_-induced lung inflammation.

## 3. Discussion

Pulmonary toxicity by PM_2.5_ is closely related to the imbalance between autophagy and inflammation by apoptosis [20]. Therefore, modulating the balance between apoptosis and autophagy may be a therapeutic and preventive strategy for lung diseases. Although CSL can protect the respiratory tract, its effect on PM_2.5_-induced respiratory diseases has been sparingly investigated. Here, CSL ameliorated autophagic dysfunction and reduced lung inflammation and apoptosis by activating the mTOR signaling pathway in an animal model of PM_2.5_-induced pulmonary damage. Therefore, CSL treatment may protect the lung from PM_2.5_-induced damage by controlling the TLR2,4–MyD88 and mTOR–autophagy pathways.

PM induces local lung inflammation by increasing the inflammatory responses of epithelial cells, endothelial cells, and macrophages [21,22,23], and systemic inflammation can occur when inflammatory mediators are overexpressed [24]. Therefore, CSL exposure may cause vascular inflammation as a biological response. Previous studies have demonstrated the relationship between PM_2.5_ exposure, reduced vascular integrity, and the expression of inflammation-associated molecules, including IL-6, TNF-α, p38, and reactive oxygen species (ROS) [25,26]. Our study established that CSL alleviated PM_2.5_-induced lung injury in a mouse model by suppressing both inflammatory cytokine secretion and lung tissue infiltration. The anti-inflammatory pathway of CSL against PM_2.5_ appears to be regulated by decreased TLR2, TLR4, and MyD88 expression, increased mTOR phosphorylation, and autophagy prevention.

An injection of 10 mg/kg PM_2.5_ caused pulmonary damages and inflammation in vivo. In previous studies, intraperitoneal PM_2.5_ injection caused respiratory and cardiovascular dysfunction by inducing systemic and local acute inflammation and stimulating histo-pathological and functional changes in mouse lung tissue [25,27,28]. In this study, PM_2.5_ was administered by intratracheal instillation, which is one of the leading methods of exposing animal models to PM_2.5_ and is usually performed by inserting a needle into the mouth and throat of mice and hamsters. Intratracheal PM_2.5_ instillation reportedly causes pulmonary injury by inducing alveolar epithelial dysfunction, inflammatory responses, and high levels of lung vessel permeability [29,30]. Although intratracheal instillation has some drawbacks, such as its non-physiological and invasive nature, the disturbing effect of anesthesia and means of delivery [31], this method is still used effectively and conveniently as only one injection induces pulmonary injury in mice [32].

Autophagy, a lysosomal-dependent process, gathers unnecessary or dysfunctional components in autophagic fluid for destruction [11]. During this process, autophagy is involved in pulmonary injury pathogenesis [33]. The activation of LC3 II, an autophagy-associated protein, in lung tissue is inhibited during mTOR activation [34], and mTOR inhibition is accompanied by upregulated LC3 II in human bronchial epithelial cells [35]. In addition, MyD88 or TLR4 knockdown downregulates lipopolysaccharide (LPS)-induced mTOR phosphorylation. These results suggested that LPS could inhibit autophagy activity, and the TLR4 signaling pathway could trigger mTOR activation [34]. Thus, despite the anti-inflammatory effects of autophagy, mTOR downregulation by rapamycin may not be effective in suppressing pulmonary injury. Therefore, autophagy and TLR4 can interact during PM-induced inflammatory responses, and autophagy can be manipulated by multiple signal transduction pathways. Moreover, TLR4 can function as an autophagy sensor involved in the PM-induced immune response [12,36]. Both TLR4–MyD88 and mTOR–autophagy pathways affect lung injury, and mTOR serves as a critical marker of autophagy in PM-induced pulmonary inflammation [35]. PM-induced inflammatory responses regulate cytokine and oxidant production through the TLP–MyD88 signaling pathway [36]. Some cytokines or oxidizing agents inhibit mTOR activation, induce cellular autophagy, and cause increased levels of tissue damage and inflammation [37]. Other signaling pathways, such as PI3K-Akt pathway [38], which regulates cell growth and survival and reduces cardiomyocyte death [39], may also control autophagy.

PI3K-Akt pathway activation reportedly phosphorylates mTOR, a vital autophagy regulator [38]. Phosphorylated mTOR prevents lung injury by reducing autophagy and promoting lung recovery [40,41]. In this study, p-mTOR, p-Akt, and p-PI3K expression were significantly recovered, and Beclin 1 and LC3 II expression were decreased by CSL. Thus, CSL suppressed excessive autophagy by upregulating the mTOR–PI3K–Akt pathway. Western blot analysis results also indicated that CSL ameliorated the PM_2.5_-induced increase in TLR 2, 4, and MyD88 expression (Figure 2). These results further indicate that CSL activates mTOR expression by reducing inflammatory cytokines and enhancing anti-inflammatory cytokines (Figure 4). Although CSL exhibited no anti-inflammatory effect, it suppressed PM_2.5_-induced lung injury by modulating PM_2.5_-mediated severe inflammatory response and autophagy compared to that in the control group. The pathway analysis suggests that CSL acts as an anti-inflammatory agent because it controls both TLR 2, 4–MyD88, and mTOR autophagy pathways.

In conclusion, CSL protects the lung from PM_2.5_-induced respiratory diseases through autophagy and TLR 2 and 4 pathway modulation. Therefore, we suggest that CSL is a potentially efficient treatment option for PM_2.5_-induced pulmonary damage.

## 4. Materials and Methods

### 4.1. Materials

Diesel PM_2.5_ NIST 1650b [42], CSL, and dexamethasone (DEX, positive control) were obtained from Sigma-Aldrich Inc (St. Louis, MO, USA). PM_2.5_ was blended in saline and sonicated for 24 h to break up the suspended particle agglomerates.

### 4.2. Animal Experiments

Seven-week-old BALB/c mice were purchased from Orient Bio Co (Seongnam, Korea). After acclimatization for 12 days, five mice were housed per cage and conditioned to a temperature of 20–25 °C. The mice experiments were conducted in accordance with the Care and Use of Laboratory Animals by Kyungpook National University (IRB No. KNU 2017-102). Mice were divided into 7 groups of 10 mice each depending on the treatment conditions: (1) control (dimethyl sulfoxide; DMSO) group, (2) CSL control group (200 μg/kg), (3) PM_2.5_ group, (4–6) PM_2.5_ + CSL group (50, 100, and 200 μg/kg), (7) PM_2.5_ + DEX group (5 mg/kg). An equivalent dose of DMSO was administered to the control group. Briefly, 10 mg/kg PM_2.5_ in 50 μL saline was intranasally injected, and CSL and DEX were administered intravenously in the tail 30 min later, as described previously [43,44]. The mice were euthanized 24 h after compound injection. Subsequently, bronchoalveolar lavage fluid (BALF) was collected for analysis. Nasal PM_2.5_ injection causes high levels of pulmonary vascular permeability and diseases, including pulmonary inflammation and alveolar epithelial dysfunction [30,45]. Therefore, this method conveniently and effectively induces lung damage with PM_2.5_.

### 4.3. Lung Wet/Dry (W/D) Weight Ratios

W/D ratio was identified to determine pulmonary edema. The weights of the right lung before and after drying in a 120 °C oven for 24 h were measured as wet and dry values, respectively.

### 4.4. Hematoxylin and Eosin (H&E) Staining

To observe phenotypic changes, the mice lungs were isolated and washed thrice with PBS at pH 7.4. Subsequently, it was fixed in 4% formaldehyde in PBS (Junsei, Tokyo, Japan) at 4 °C for 20 h. The fixed samples were embedded, in paraffin, dehydrated, and cut into 4-µm sections. Next, the samples were deparaffinized, rehydrated, and stained with hematoxylin (Sigma-Aldrich, St. Louis, MO, USA). The lung specimens were visualized under a light microscope to monitor the lung structure and tissue edema, as described previously [46,47].

### 4.5. Enzyme-Linked Immunosorbent Assay (ELISA)

Bax, Bcl-2 (LifeSpan BioScience, Inc.; Seattle, WA, USA), truncated-PARP, TNF-α, IFN-γ, IL-1β, IL-2, IL-4, IL-6, IL-8, IL-10, IL-18 (R&D Systems; Minneapolis, MN, USA), cleaved caspase-3, and cleaved PARP expression were quantified using a commercially available ELISA kits, according to the manufacturer's instructions. All analyses were performed on the Tecan plate reader (Tecan Austria GmbH; Grödig, Austria).

### 4.6. Cell Counts in BALF Samples

The BALF samples were centrifuged at 3000 rpm for 10 min at 4 °C before measurement, and the cells were analyzed using a blood analyzer. Whole cells were stained with anti-CD11b monoclonal antibody (M1/70), FITC, eBioscience™ (Thermo Fisher, 11-0112-41), and leukocytes were stained with anti-CD45 monoclonal antibody (HI30), FITC, eBioscience™ (Thermo Fisher, 11-0459-42) leukocyte staining) at 4 °C for 2 h. Subsequently, the cells were washed twice with PBS, re-suspended in 1 mL PBS, and the fluorescence was quantified using a FACScan flow cytometer (BD).

### 4.7. Western Blotting

Lysed sample using lysis buffer containing 1% NP-40, 1% sodium deoxycholate, 0.5% sodium dodecyl sulfate (SDS), protease inhibitor, 50 mM Tris-HCl (pH 7.5), and 150 mM NaCl was added, as previously described [43,44]. The membranes were blocked in 5% BSA for 2 h and incubated with the primary antibodies against light chain (LC)3 (1:1000), Beclin 1 (1:1000), TLR2 (1:1000), TLR4 (1:1000), mTOR (1:1000), MyD88 (1:1000), phosphorylated (p)-mTOR (1:1000), Akt (1:1000), p-Akt (1:2000), p-PI3K (1:1000), and PI3K (1:800) (all Cell Signaling Technology, Inc.). After washing the membrane, horseradish peroxidase (HRP)-conjugated secondary antibody was added and incubated (Cell Signaling Technology, 1:10,000). Subsequently, the ImageJ Gel Analysis tool was used to perform the concentration analysis (NIH; Bethesda, MD, USA).

### 4.8. Terminal Deoxynucleotidyl Transferase dUTP Nick End Labeling (TUNEL) Assay

The mesenchymal of the right lung was used to detect apoptotic cells in lung tissue using the TUNEL assay. The TUNEL reaction mixture (Roche Applied Science; Mannheim, Germany) was prepared and the total apoptotic cells was shown as the % of TUNEL-positive cells to the total cells by Hoechst staining.

### 4.9. Statistical Analysis

The experiments were repeated at least thrice and performed independently. The data are expressed as the mean ± standard deviation (SD). SD values were analyzed using a one-way analysis of variance (ANOVA) followed by Dunnett’s test. All analyzes were performed using SPSS for Windows version 16.0, and *p* < 0.05 were considered statistically significant (SPSS, Chicago, IL, USA).

## Data Availability

The data presented in this study are available upon reasonable request from the corresponding author.
